# Breaking the Invisible Barriers: Unleashing the Full Potential of Immune Checkpoint Inhibitors in Oncogene-Driven Lung Adenocarcinoma

**DOI:** 10.3390/cancers15102749

**Published:** 2023-05-13

**Authors:** Hoi-Hin Kwok, Jiashuang Yang, David Chi-Leung Lam

**Affiliations:** Department of Medicine, Li Ka Shing Faculty of Medicine, The University of Hong Kong, Hong Kong SAR, China; kwokh@hku.hk (H.-H.K.); jsyang@connect.hku.hk (J.Y.)

**Keywords:** non-small cell lung cancer, targeted therapy, immune checkpoint inhibitor, tumor immune microenvironment

## Abstract

**Simple Summary:**

The development of targeted therapies has led to personalized medicine for advanced non-small cell lung cancer (NSCLC), particularly in lung adenocarcinoma (ADC) with actionable genetic alterations, such as *EGFR*, *ALK*, *KRAS*, and *ROS1*. Tyrosine kinase inhibitors (TKIs) and angiogenesis inhibitors have shown better therapeutic responses and lower toxicity compared to systemic chemotherapy. However, resistance to these therapies remains a challenge. Immune checkpoint inhibitors (ICIs) targeting PD-1 or PD-L1 have changed the treatment paradigm for NSCLC without actionable genetic alterations, but combining targeted therapy and ICIs does not provide survival benefits. The review focuses on the tumor immune microenvironment and its role in applying ICIs for this subpopulation of lung ADC patients.

**Abstract:**

The rapid development of targeted therapy paved the way toward personalized medicine for advanced non-small cell lung cancer (NSCLC). Lung adenocarcinoma (ADC) harboring actionable genetic alternations including epidermal growth factor receptor (*EGFR*), anaplastic lymphoma kinase (*ALK*), Kirsten rat sarcoma virus (*ALK*) and c-ros oncogene 1 (*ROS1*) treated with tyrosine kinase inhibitors (TKIs) incurred lesser treatment toxicity but better therapeutic responses compared with systemic chemotherapy. Angiogenesis inhibitors targeting vascular endothelial growth factor (VEGF) have also shown an increase in overall survival (OS) for NSCLC patients. However, acquired resistance to these targeted therapies remains a major obstacle to long-term maintenance treatment for lung ADC patients. The emergence of immune checkpoint inhibitors (ICIs) against programmed cell death protein 1 (PD-1) or programmed cell death-ligand 1 (PD-L1) has changed the treatment paradigm for NSCLC tumors without actionable genetic alternations. Clinical studies have suggested, however, that there are no survival benefits with the combination of targeted therapy and ICIs. In this review, we will summarize and discuss the current knowledge on the tumor immune microenvironment and the dynamics of immune phenotypes, which could be crucial in extending the applicability of ICIs for this subpopulation of lung ADC patients.

## 1. Introduction

In the last century, surgery, radiotherapy and chemotherapy were almost the only therapeutic options for the treatment of lung cancer. The median overall survival (mOS) for the disease remained as low as a few months. Nowadays, with the advances in targeted therapy and immunotherapy, the mOS of advanced non-small cell lung cancer (NSCLC) leaps to more than a year [1]. However, lung tumors that are not responsive or even resistant to current treatment remain an unresolved issue. For NSCLC patients with a druggable oncogene, such as tumor-detected epidermal growth factor receptor (*EGFR*), anaplastic lymphoma kinase (*ALK*), Kirsten rat sarcoma virus (*KRAS*) and c-ros oncogene 1 (ROS1), tyrosine kinase inhibitors (TKIs) are primarily recommended as first-line treatment. On the other hand, for NSCLC patients whose tumors lack druggable driver alternations, immunotherapy in the form of immune checkpoint inhibitors (ICIs) has emerged as a durable treatment option for patients with high tumor programmed cell death—ligand 1 (PD-L1) expression. However, subgroup analysis of various clinical studies has shown that druggable-mutation-positive NSCLC may have an inferior response to first- or second-line ICIs treatment [2,3], with the relevant mechanisms remaining largely unknown. Here, we provide a concise review of current knowledge on the tumor immune microenvironment (TIME), which may be crucial to potentiate the use of ICIs for this subgroup of NSCLC patients.

## 2. Tyrosine Kinase Inhibitors and Immune Checkpoint Inhibitors for Advanced Lung Adenocarcinoma

### 2.1. Tyrosine Kinase Inhibitors

The rapid development of tyrosine kinase inhibitors (TKIs) in the past two decades drove toward precision medicine with the advancement of molecular diagnostic tools [4]. By inhibiting specific receptor protein kinase, which their gene mutation or overexpression is essential for cancer cell proliferation, targeted therapy can induce regression of tumors more effectively and with lesser side effects when compared with conventional chemotherapy. Further genomic research identified additional oncogenes that also contributed to the development of NSCLC. The prevalence of different oncogenic driver mutations in NSCLC varies between Caucasian and Asian countries [5]. In Asia, a major subpopulation of NSCLC patients harboring certain druggable oncogenes, including epidermal growth factor receptor (*EGFR*) (~50%) and a smaller proportion with Kirsten rat sarcoma virus (*KRAS*) mutations (~10–20%), anaplastic lymphoma kinase (*ALK*) (~5%) or ROS proto-oncogene 1 (*ROS1*) rearrangement (<5%). *KRAS* mutations, however, could be found in about 30% of NSCLC in Caucasian populations. Indeed, some genetic variants are more common in certain geographic regions, such as *TP53* mutations are higher in Iranian patients with lung cancer [6]. NSCLC patients with tumor-bearing druggable mutations usually show a good initial response to respective targeted therapy, but most will eventually progress in one to two years due to acquired drug resistance. Newly developed next-generation TKIs, including osimertinib for *EGFR* mutations, lorlatinib and brigatinib for *ALK* re-arrangement, sotorasib and adagrasib for *KRAS* G12C mutations, and entrectinib for *ROS1* rearrangement, are currently US-FDA approved next-line treatment for NSCLC patients with respective resistance following primary therapy. However, selection pressure from specific targeted therapy can induce gradual genetic and epigenetic adaptation in heterogenous clones of cancer cells, leading to an enrichment of the best-fit subpopulation of cancer cells within the tumor microenvironment [7]. However, most of the research deconvolute acquired resistance to a single identifiable origin, such as point mutation (*EGFR* T790M, *ALK* L1196M) or gene amplification (*MET*). Such an assumption of a single hit resistance mechanism may allow for the further emergence of unidentified mechanisms of drug resistance. The need to search for long-lasting and low-toxicity treatment strategies remains.

Besides oncogenic mutations, another significant therapeutic approach involves directing treatment toward growth factors that promote tumor angiogenesis. By sprouting new vasculature towards the tumor mass to sustain its active metabolism and metastasis, tumor angiogenesis is essential for tumor growth and metastasis [8]. Cancer cells can induce tumor angiogenesis by releasing pro-angiogenic growth factors, including vascular endothelial growth factor (VEGF), fibroblast growth factor (FGF), and platelet-derived endothelial cell growth factor (PDGF) [9]. A variety of angiogenesis blockade therapies in combination with other chemotherapy have been approved for use in different advanced-stage cancers [10]. Bevacizumab is the first FDA-approved VEGF-targeted monoclonal antibody for NSCLC. These anti-angiogenic agents are under study in conjunction with ICIs or other chemotherapies with the speculation they may help to alleviate the induction of drug resistance [11]. Results from recent clinical trials, however, revealed significant potentiation of immune-related adverse effects (irAEs), which may limit the applications of anti-angiogenic agents in combination with ICIs [12].

### 2.2. Immune Checkpoint Inhibitors

The development of immunotherapy, especially immune-checkpoint inhibitors (ICIs), rapidly emerged as the cornerstone of the management of NSCLC. Tumor mutations lead to aberrant protein-coding that eventually results in the expression of tumor antigens, which could be recognized, processed and presented by major histocompatibility complexes (MHC) on antigen-presenting cells (APCs), such as dendritic cells (DCs), to cytotoxic T cells in lymph nodes. Primed and activated T cells will then seek and kill tumor cells by recognizing tumor antigens. Although tumor cells can be eliminated by the immune system, certain subpopulations of tumor cells manage to escape immunosurveillance by the body. Indeed, for recognition of tumor cells by effector T cells, programmed cell death protein 1 (PD-1) is critical in counteracting the tumor immune responses. Upon binding with programmed death-ligand 1 (PD-L1) on tumor cells, PD-1 serves as a negative signal which normally keeps T cells from attacking normal cells. Some cancer cells can express high levels of PD-L1, which supports their escape from immune attack. ICIs targeting PD-1 and PD-L1 reinvigorate T cells that were inactivated by the PD-1/PD-L1 signaling. It has been proven that PD-1/PD-L1 blockade therapy significantly stimulates the anti-tumor effects of cytotoxic T lymphocytes in different solid tumors, including advanced-stage lung cancer. Clinically, first-line ICIs treatment attained a mOS of 16.2 months and 50% of 2-year survival in non-oncogene-driven NSCLC patients [13]. However, only 20% of NSCLC patients achieve this durable objective response.

In recent years, tumor PD-L1 expression (tumor proportional score [TPS]) has been the most commonly used predictive biomarker for anti-PD-1 treatment. A PD-L1 TPS level of higher than 50% in tumor cells is regarded as the most promising biomarker for anti-PD-1 and anti-PD-L1 treatments in those patients who could benefit from immunotherapy. In KEYNOTE-024, pembrolizumab prolonged primary endpoint, median progression-free survival (PFS), and median overall survival (OS) compared with platinum-based chemotherapy in 305 patients with PD-L1 TPS score of ≥50% [14]. KEYNOTE-042 showed that pembrolizumab significantly improved OS compared with chemotherapy (median OS 16.7 months vs. 12.1 months) in patients with high PD-L1 expression (TPS ≥ 50%). However, in patients with relatively lower levels of PD-L1 expression (TPS = 1–49%), the OS achieved with pembrolizumab or chemotherapy was similar (median OS 13.4 vs. 12.1) [15]. Due to the promising efficacy of pembrolizumab, the US Food and Drug Administration (FDA) approved it as the first-line therapy for PD-L1 ≥ 1% tumor cells, and European Medicines Agency (EMA) approved it for tumors with PD-L1 expression ≥ 50%. Recently, IMPOWER-Lung 01 trial and IMpower-110 demonstrated that cemiplimab (an anti-PD-1 antibody) and atezolizumab (an anti-PD-L1 antibody) could improve OS effectively compared with platinum-based chemotherapy. FDA and EMA approved atezolizumab as first-line therapy for NSCLC patients with TPS ≥ 50%. Atezolizumab has been approved as monotherapy for high PD-L1-expressing NSCLC patients [16]. Other biomarkers, such as tumor mutational burden (TMB), defined as a tumor with TMB ≥ 10 mutation/megabase, have been approved recently that showed a higher frequency of response to ICIs therapy in the advanced solid tumor [17]. Other emerging biomarker assays, including multiplex immunofluorescence assay, could yield even better predictive values [18], which suggested there is room for improvement on predictive biomarkers with an improved understanding of the TIME.

## 3. Clinical Hurdles in Combining TKIs and ICIs for Treatment in NSCLC

The combination of TKIs and ICIs remains an attractive treatment strategy to explore. Results from the CheckMate057 and KEYNOTE-010 demonstrated a statistically significant improvement in the OS in NSCLC patients treated with nivolumab or pembrolizumab in comparison to patients receiving standard second-line docetaxel-based chemotherapy [19,20]. However, these studies also showed that *EGFR*-mutated NSCLC patients did not derive benefit from using immunotherapy compared with chemotherapy. In KEYNOTE-010, 86 patients (8.3%) were *EGFR*-mutant, and 6 (0.6%) were *ALK*-positive; patients with these oncogenic driver mutations did not achieve prolonged OS in response to pembrolizumab compared to docetaxel. In CheckMate057, 82 patients (14% of all) were *EGFR*-mutant, and 21 (4%) were *ALK*-positive. Subgroup analyses of OS revealed that patients with *EGFR* mutation, having received or received an additional line of TKI, did not benefit from nivolumab compared with docetaxel (hazard ratio [HR] 1.18, 95% confidence interval [CI]: 0.69–2.00).

Early studies suggested that *EGFR* mutations in NSCLC could induce up-regulation of PD-1 expression through activation of the ERK signaling pathway, which may mediate the immune escape [21,22]. Further meta-analysis revealed that there was no significant association between PD-L1 expression and OS in NSCLC treated with EGFR-TKI, suggesting that ICIs should only be considered after other effective therapies have been exhausted in *EGFR*-mutated lung tumors [23]. The heterogeneity of study findings on *EGFR* mutation status and PD-L1 expression suggested the indirect and complicated correlation between the two factors [24]. Indeed, the expression of PD-L1 can also affect the therapeutic efficacy of EGFR-TKI [25], indicating the potential crosstalk between the EGFR pathway and PD-L1 expression in tumors. Similarly, previous studies demonstrated the interactions between the PD-L1 and EML4-*ALK* fusion protein [26] and *KRAS* mutations [27] in NSCLC. Both clinical and molecular studies strongly suggested that PD-L1 expression has an independent impact on sensitivity to ICIs for the oncogene-driven advanced lung ADC [28]. These observations in both laboratories and clinics suggested that further understanding of the tumor immune microenvironment (TIME) of oncogene-driven NSCLC is needed to elucidate this phenomenon and may potentially shed light on new treatment strategies.

## 4. Dynamics of Tumor Immune Cycle in the Tumor Microenvironment

The battling of immunity against malignant cells is a tightly regulated multi-step process, which is determined by the counteracting and highly dynamic effects of different immune cells, along with their interactions and responses to the malignant cells. The tumor immune cycle proposed by Daniel Chen and Ira Mellman has become the conceptual framework in cancer immunotherapy research [29].

The framework described that persistent accumulation of DNA damage in a cell that is not properly repaired could lead to genomic instability, which may affect normal cellular functions and ultimately lead to malignant transformation. The unrepaired DNA generates neoantigens, which can be recognized by antigen-presenting cells (APC) through major histocompatibility class I (MHC-I) molecules on malignant cells. These neoantigens released by malignant cells could be recognized by the immune system. Dead and dying malignant cells release neoantigens and damage-associated molecular patterns (DAMPs, e.g., extracellular matrix and intracellular compartments, such as heat-shock proteins, ATP, and histones molecules) that are captured by and activate conventional dendritic cells. Activated dendritic cells travel back to lymph nodes where they process and present MHC-I-bound-neoantigens to naïve CD8^+^ T cells through the binding of T cell receptors (TCRs), along with B7-CD28 binding, which results in full activation of CD8^+^ T cells [30]. Activated tumor-specific effector T cells migrate from the lymph nodes and infiltrate into the tumor bed, where TCRs on T cells bind to MHC-I-bound neoantigens on malignant cells. The effector T cells secrete more IFN-γ, which binds to IFN-γ receptors on nearby tumor and normal cells, resulting in further increased MHC-I antigen presentation in those cells and enhancing additional TCR engagement and cytotoxic activity to malignant cells. At the same time, effector T cells eradicate malignant cells through apoptotic signaling via Fas/FasL, secretion of granzyme and perforin, and direct lysis of tumor cell membrane, resulting in the release of extra tumor-associated antigens and cytokines that sustain tumor immune response in successive tumor immune cycles [31]. Part of the effector T cells will also differentiate into memory T cells, which can recognize and respond more quickly to the same tumor neoantigens in a future encounter.

The tumor immune cycle is a dynamic process that can be disrupted by various mechanisms, such as immune evasion by tumor cells, inhibition of T cell activation, or suppression of immune responses. In NSCLC patients who respond effectively to ICIs therapy, precise execution of tumor immune cycles by restoring harmony to the immune system is typically noted. This observation also indicates that low immunogenicity and insensitivity to ICI in oncogene-driven lung ADC suggested the presence of unknown barriers during the tumor immune cycle.

### 4.1. Role of Different Immune Cells in TIME

The dynamic interactions between tumor and immune cells determine the development of tumor immunity and response to treatments. Recent studies have shown that cancer cells, particularly in oncogene-driven lung ADC, can affect their normal counterparts to create an immunosuppressive TIME. The different cell types within the TIME engage in reciprocal communication that involves a network of growth factors, cytokines, extracellular vesicles, and adhesion molecules. These complex interactions play fundamental roles in promoting tumor progression and can significantly affect the response of tumors to ICIs. The rapid growth of high-throughput single-cell sequencing technologies has resolved the detailed varieties of subpopulations of immune cells, which allows for substantial improvement in the characterization of diverse immune cells [32].

#### 4.1.1. Lymphoid Cells

##### CD8^+^ Cytotoxic T Cells

CD8^+^ cytotoxic T lymphocytes (CTLs) play a crucial role in the tumor immune cycle by recognizing and eliminating cancer cells. Upon activation through the engagement of TCR on CD8^+^ T cells with neoantigens presented by MHC-1 on APCs and target cells, followed by secondary activation signal by CD28 and CD80 co-stimulatory signals, CD8^+^ T cells can directly kill tumor cells by releasing cytotoxic molecules, such as perforin and granzyme B. Perforin is a cytolytic pore-forming protein which allows entry of granzyme serine protease to activate the caspase cascade and eventually induces apoptosis of target cells. However, CD8^+^ T cells can also have negative regulatory roles in the tumor immune cycle. For instance, they can become terminally exhausted (progenitor-exhausted), which could limit their ability to recognize and kill tumor cells, but also actively participate in tumor progression by maintaining the cancer stemness [33]. This occurs due to prolonged exposure to the tumor antigens [34] or chronic inflammation [35], or inhibitory signals released from the TIME [36]. However, a study from Nishii et al. demonstrated that CD8^+^ T cell responses were induced by EGFR-TKI. They showed that EGFR inhibition is a prerequisite for the subsequent ICI treatment [37].

##### CD4^+^ Helper T Cells

CD4^+^ helper T cells are important regulatory cells providing help to other immune cells, including CD8^+^ T cells, to mount an effective anti-tumor response. A recent study showed that CD4^+^PD-1^+^CXCL13^+^ cells (or previously classified as putative T_FH_ cells) are direct targets of anti-PD-1 antibodies, which act as a potential hub for APCs in the TIME [38]. CD4^+^ T cells can differentiate into different subsets, including Th1, Th2, Th17, and Treg cells, that have distinct functions in tumor immunity. Th1 cells produce cytokines, such as IFN-γ, that activate CD8^+^ T cells and promote cytotoxicity against tumor cells [39,40]. They can also activate macrophages [41] and interact with tumor-infiltrating B cells to induce anti-tumor activity [42]. Th2 cells, which are a key player in allergic asthma, were also abundantly found in NSCLC [43]. They produce cytokines, such as interleukin-4 (IL-4) and interleukin-13 (IL-13). These cytokines, in turn, promote the proliferation of myeloid-derived suppressor cells and suppress cytotoxicity against tumor cells [44]. Th17 cells produce cytokines, such as interleukin-17 (IL-17) and interleukin-22 (IL-22), that recruit neutrophils and promote inflammation. The role of Th17 in tumor development remains controversial [45]. Salazar et al. showed that Th9 and Th17 cells induce metastasis in lung cancer [46]. Preclinical experiments by Peng et al. also demonstrated that a triple combination of inhibition of IL-17, MEK and PD-L1 could overcome resistance to combined MEK and PD-L1 inhibitor [47]. Regulatory T cells (Treg), which are characterized by the expression of transcription factor FoxP3 and surface CD25, have a suppressive function and can inhibit the activity of other immune cells, including CD4^+^ and CD8^+^ T cells. They play a critical role in preventing autoimmunity and maintaining self-tolerance but can also inhibit effective anti-tumor immunity. Co-treatment of IL-6 with anti-PD-1 could enhance the activation of tumor-infiltrating Treg and Th17 in EMT-associated TKI-resistant *EGFR*-mutant NSCLC [48]. Therefore, CD4^+^ helper T cells can have both positive and negative regulatory roles in the tumor immune cycle, depending on their subset and the context of the tumor microenvironment [49].

##### Tumor-Infiltrating B Cells

Although T cells remain the focus in immune-oncology, increasing evidence suggests that intratumoral or peritumoral B cells are associated with the response to immunotherapy. B cells in many tumors are often organized in tertiary lymphoid structures (TLS). The significance of active versus inactive TLS remains a key open question [50]. Tumor-infiltrating B cells (TIBs) can play both positive and negative regulatory roles in the tumor immune cycle [51]. TIBs can contribute to the positive regulation of the tumor immune cycle by producing antibodies against neoantigens [52]. These antibodies can help to activate other immune cells, such as CTLs, which can recognize and kill cancer cells [53]. TIBs can also act as APCs, presenting neoantigens to CTLs and promoting their activation [42]. Additionally, TIBs can produce cytokines that promote the recruitment and activation of other immune cells, such as macrophages and dendritic cells [54]. On the other hand, TIBs can also play a negative regulatory role in the tumor immune cycle. Some tumors can promote the differentiation of B cell precursors to generate macrophage-like cells, which suppress the proliferation of T cells and induce FoxP3^+^ Treg [55]. IL-35-producing B cells can also produce immunosuppressive cytokines, such as IL-10 and TGF-β, which further inhibit the activity of CTLs and other immune cells [56]. TIBs can also express immune checkpoint molecules, such as PD-L1, which can bind to PD-1 receptors on CTLs and inhibit their activity [57]. This may result in the suppression of immune response against cancer cells and promote tumor growth.

##### Natural Killer (NK) Cells

NK cells are capable of recognizing and killing cancer cells without the need for prior sensitization or activation, making them an important first line of defense against cancer. NK cells can recognize and directly kill cancer cells through the recognition of stress-induced ligands on the surface of cancer cells, which are up-regulated in response to cellular stress. These ligands can bind to the activating receptor NKG2D on NK cells, leading to the activation and killing of cancer cells [58]. NK cells can also identify and kill cancer cells coated with antibodies, a process known as antibody-dependent cell-mediated cytotoxicity (ADCC) [59]. Chronic activation of NK cells causes the expression of immune checkpoint receptors, including PD-1, CTLA4, TIM3, and LAG3, which explains the progressive dysfunction of NK cells during the progression of cancer [60].

#### 4.1.2. Myeloid Cells

##### Tumor-Associated Macrophage (TAM)

Cumulative evidence suggested that TAM are key determinant of T cell excluded tumor phenotypes observed in oncogene-driven NSCLC [61,62]. However, it is worth noting that the polarization of macrophages into tumor-suppressive M1 or tumor-promoting M2 types is a fundamental event in the establishment of TIME [63]. Pro-inflammatory M1 macrophages are characterized by their ability to produce high levels of pro-inflammatory cytokines, reactive oxygen species (ROS), and nitric oxide (NO). M1 macrophages also have enhanced antigen-presenting capabilities and can promote the activation of T cells against cancer cells [64]. On the other hand, M2 macrophages are activated by anti-inflammatory cytokines, such as IL-4 and IL-13, and are characterized by their ability to produce high levels of anti-inflammatory cytokines, such as IL-10 and TGF-β. M2 macrophages also have enhanced tissue repair and immunomodulatory capabilities and can promote angiogenesis, which can support tumor growth [65]. However, a high M1:M2 density ratio in the tumoral area was associated with better cancer-specific survival [66]. Macrophage polarization state, rather than their overall density, was associated with cancer-specific survival, with M1- and M2-like macrophage phenotypes exhibiting distinct prognostic roles [67].

##### Tumor-Associated Neutrophils (TAN)

TAN could be recruited to the tumor microenvironment in response to inflammatory signals, but the role of TANs in tumor immunity is complex and can be both pro-tumorigenic and anti-tumorigenic depending on the context [68]. TANs have been shown to promote tumor growth and metastasis by several mechanisms, including the release of growth factors, such as vascular endothelial growth factor (VEGF), which can promote angiogenesis and support tumor growth, and the production of matrix metalloproteinases (MMPs), which can degrade the extracellular matrix and facilitate tumor invasion and metastasis [69]. Additionally, TANs in NSCLC can produce high levels of interleukin-17 (IL-17), which can promote tumor growth and metastasis by inducing angiogenesis and suppressing the activity of T cells [70]. However, some studies showed that TANs could directly kill NSCLC cancer cells through the release of ROS and the formation of neutrophil extracellular traps (NETs) [71]. Additionally, comprehensive immune cell profiling in lung cancer revealed that the immune cell composition is fundamentally different in lung adenocarcinoma as compared with lung squamous cell carcinoma and that neutrophils are the most prevalent immune cell type, suggesting therapeutic manipulation of neutrophils may enhance ICI responsiveness [72].

##### Myeloid-Derived Suppressor Cells (MDSCs)

MDSCs are a heterogeneous population of immature myeloid cells that can suppress the immune response and promote tumor growth [73]. MDSCs can produce immunosuppressive molecules, such as arginase, nitric oxide, and reactive oxygen species (ROS), which can inhibit the activity of T cells in the NSCLC [74]. MDSCs can also promote the expansion of regulatory T cells (Tregs), which can further suppress the anti-tumor immune response [75]. The number of circulating MDSCs in NSCLC has been associated with poor prognosis and resistance to immunotherapy [76].

##### Dendritic Cells (DC)

DC are specialized antigen-presenting cells that can capture and process antigens from tumor cells and present them to T cells, initiating an immune response against cancer cells [77]. Conventional DC (cDC) is a rare but important subset among all DC. Tumor-resident cDC1s are the predominant sources of the T-cell chemokines supporting the recruitment of effector T cells into the tumor microenvironment [78]. In addition to their role in antigen presentation, DCs can also modulate the tumor microenvironment by producing cytokines and chemokines that attract other immune cells to the site of the tumor [79]. DCs can also promote the differentiation of effector T cells and memory T cells, which can provide long-lasting protection against cancer cells [80].

##### Mast Cells

The role of mast cells in tumor development remains controversial in NSCLC [81]. Mast cells can promote tumor growth and progression by releasing pro-inflammatory mediators and growth factors that stimulate angiogenesis and tumor cell proliferation [82]. Mast cells can also suppress the anti-tumor immune response by inhibiting the activity of T cells and natural killer cells [83]. It has been shown that a high frequency of total tumor-associated mast cells is associated with better overall survival and progression-free survival in NSCLC patients [84].

### 4.2. Tumor Immune Phenotypes

According to the spatial distribution relative to the tumoral area and the activation status of immune cells, as mentioned above, there are three recognized sub-types of tumor immune phenotypes, immune-deserted tumor, immune-excluded tumor, and immune-enriched tumor.

#### 4.2.1. Immune-Deserted Tumor

Immune-deserted tumors are a specific tumor immune phenotype category characterized by a lack of tumor-infiltrating lymphocytes (TILs) in both the tumor and stromal areas and low antigen presentation. This phenotype can result from several factors, including a low gradient of chemokines required for dendritic cell recruitment [85]. The altered metabolic landscape of the tumor microenvironment may also inhibit the infiltration of various immune cells [86]. It has been observed that NSCLCs with inactivating mutations in *LKB1* exhibit a poor response to anti-PD1 therapy despite a high tumor mutational burden (TMB) due to reduced expression of the immunoproteasome, impairing antigen presentation. However, a lower proteasome activity can lead to enhanced autophagy as a compensatory mechanism. Inhibition of autophagy by targeting ULK1 has been shown to restore antigen presentation and synergize with PD-1 antibody blockade, promoting tumor regression in LKB1-mutant mice. [87]. Different approaches, including radiotherapy, chemotherapy and virus infection, are proposed in order to induce immunogenic cell death in tumors that result in improved immune recognition for immune-deserted tumor [88].

#### 4.2.2. Immune-Excluded Tumor

Immune-excluded tumors are a tumor immune phenotype characterized by the presence of cytotoxic T cells within the tumor stroma but not infiltrating the tumor area. This phenotype can result from several factors, including the expression of chemokines/cytokines, such as CCL2, IL6, IL10, and TGFβ, as well as the presence of cell types associated with immune suppression or tolerance, such as Treg cells [89]. Despite a relatively high tumor mutation burden associated with NSCLC [90], it has been well-documented that oncogene-driven NSCLC is associated with uninflamed TIME with immunological tolerance and weak immunogenicity [2]. In response to ICI treatment, T cells in tumor stroma can undergo activation and proliferation but do not infiltrate the tumor area, leading to poor clinical responses. Some studies showed that NSCLC could exhibit immune-excluded phenotype due to factors such as vascular barriers, immune suppressive cancer-associated fibroblasts (CAFs), or excessive deposition of extracellular matrix. Emerging evidence suggests that inhibiting TGF-β signaling may be a promising therapeutic strategy for immune-excluded tumors [91]. A justified dose of anti-angiogenic agents can potentially normalize the tumor vasculature and reduce the presence of immune suppressive cells, leading to improved T-cell infiltration and better responses to ICIs.

#### 4.2.3. Immune-Enriched Tumor

An immune-enriched tumor phenotype is characterized by the presence of various TILs, including CD4- and CD8-expressing effector T cells, as well as inhibitory Tregs, myeloid-derived suppressor cells, suppressor B cells, and CAFs. In NSCLC, immune-enriched tumors are associated with a smoking history and a high TMB [92] but also exhibit co-occurrence with immune-excluded regions. Active effector TILs and TIL-B densities in this immune phenotype are linked to positive prognostic of NSCLC patients with ICI treatment [93]. These immune cells are found in proximity to tumor cells, with CD8^+^ cells often showing an exhausted and dysfunctional state [94]. PD-L1 staining may be observed on both infiltrating immune cells and tumor cells, and an abundance of type-I and type-II interferons, IL-12, IL-23, IL-1β, TNFα, IL-2, granzymes, CXCL9, CXCL10, and other pro-inflammatory/effector cytokines may also be observed [95]. In addition, the presence of TLS in NSCLC tumors has been linked with favorable clinical outcomes in patients receiving immunotherapy. However, the mechanisms behind TLS formation and the role of TLS in anti-tumor immunity in NSCLC remain under investigation [96]. Despite certain challenges remain, such as adverse effects and acquired resistance, anti-angiogenic agents in combination with ICIs have demonstrated promising outcomes in many types of cancers. These results indicated that lung ADC with immune-enriched tumors, even if it may not be the major case, could be beneficial in anti-angiogenic therapy [97].

### 4.3. Immunological Effect of Oncogenes and Their Specific TKIs in TIME

Oncogenic driver mutations do not just promote abnormal cell growth and division, leading to the development of tumors; in addition, oncogenes also equip tumor cells with the ability to modulate the recognition and assault by the immune system, contributing to the development of an immunosuppressive TIME that facilitates tumor growth and progression (Table 1). The effect of TKIs on the modification of TIME to the immune response is under intensive investigation. Studies showed that excessive immune cells, including T cells, B cells, macrophages, polymorphonuclear cells, mast cells, natural killer cells, dendritic cells and MDSCs, infiltrate into TIME after TKIs treatment (Table 2).

**Figure 1:**
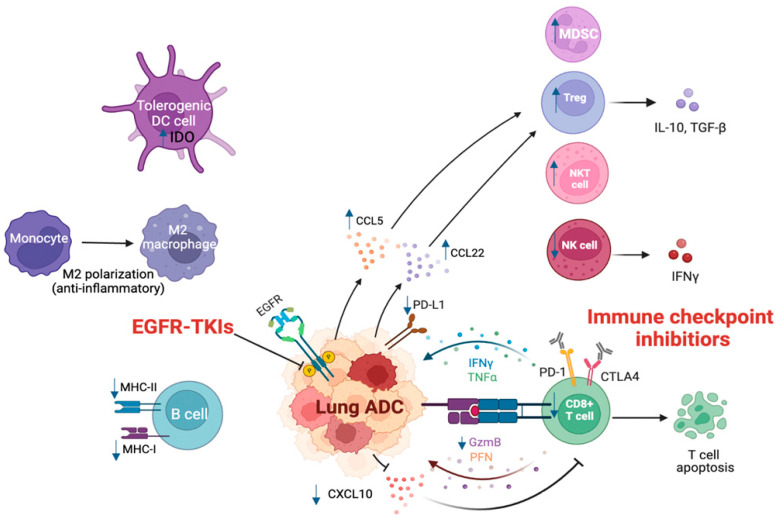
TIME in lung ADC with *EGFR* mutation.

In short-term TKIs treatment, CD8^+^T cells and DC cells expand, and T cell infiltration increases with a concomitant decrease in immunosuppressive cells, such as FoxP3^+^ Tregs and M2-like polarization of macrophages [98]. TILs and cytokine levels were transformed after EGFR-TKIs therapy [99]. After a 4-week gefitinib treatment, the number of peripheral NK cells increases, and the release of IFN-γ accumulates. Erlotinib and gefitinib can improve the killing capacity of NK cells to cancer cells [100]. Meanwhile, EGFR-TKIs can increase major MHC-I and -II molecules and induce T cell-mediated killing to tumor cells [101]. The significantly decreasing level of IL-6 indicates better progression-free survival [102]. EGFR inhibition decreases pro-inflammatory cytokines, such as CC chemokine ligand CCL2, CCL5, C-X-C motif chemokine ligand CXCL8, CXCL10, and IFN-γ-induced protein 10 (IP-10) and increases the release of IFN-γ [99]. In animal models with *EGFR* mutation, the application of erlotinib increases infiltrating T lymphocytes and NK cells other than B cells. Increasing MHC-II expression in antigen-presenting cells, such as dendritic cells and macrophages, can enhance antigen-presenting activity. TKIs can reduce the levels of immune checkpoints in TIME. Studies suggested PD-L1 expression was downregulated by inhibiting the *EGFR* pathway after 4 weeks of gefitinib treatment [103]. EGFR-TKIs treatment can also lead to a decrease in PD-1, cytotoxic T lymphocyte-associated antigen 4 (CTLA-4) and T cell immunoglobulin and mucin domain-containing protein 3 expression in T cells in mouse models [98] in NSCLC patients who failed TKIs therapy followed by drug resistance. Acquired resistance of TKIs creates an immunosuppressive TME to assist tumor cells in escaping from immunosurveillance. EGFR-TKIs resistance NSCLC cells have a lack of cytotoxic T cells in infiltration T cells and deficiency of MHC-I expression [104,105]. Treg cells are the main regulators of TIME. Macrophage expressing indoleamine 2,3-dioxygenase 1 (IDO1) increases to accelerate cancer progression [106]. The increasing percentage of suppressive MDSCs weakens the activation of T cells after long-term TKIs use. In the serum of NSCLC patients with TKIs resistance, higher MDSCs were detected than baseline. In line with EGFR mutations, a mouse model of Kras/Trp53 loss (KP)-driven lung ADC showed resistance to ICIs, which is mediated by protein kinase Cι (PKCι)-YAP signaling, and enhanced infiltration of MDSCs and decreased infiltration of CD8^+^ T cells [107]. Overall, long-term TKI treatment will influence the function of T effector cells and antigen-presenting cells, which impair immune response in the TIME [108]. More importantly, TKI treatment could significantly promote PD-L1 expression and block the killing of T cells in TIME. Blocking the PD-L1 pathway by ICIs could reduce the interaction with PD-1 in lymphocytes (Figure 1).

*EGFR*-mutated lung ADC promotes tumor development. *EGFR* mutation increases PD-L1 expression in cancer cells, which can promote the exhaustion and apoptosis of CD8^+^ T cells. As a proinflammatory chemokine, decreased CXCL10 reduces the migration and recruitment of CD8^+^ T cells and lowers the infiltration of CD8^+^ T cells. Elevated production of CCL5 and CCL22 promotes the migration of Treg cells and further attenuates the function and proliferation of CD8^+^ T cells. *EGFR* mutation also induces upregulation of MDSCs, increases IDO in DCs, and enhances M2-like polarization and migration of TAMs, which abrogates CD8^+^ T cell and cytotoxic NK cell-mediated tumor killing. *EGFR* mutation also leads to a decrease in the activation of B cells, lowering the expression of MHC-I and -II on B cells, and decreasing the activation and cytotoxicity of NK cells, while increasing cytotoxicity of NKT cells. EGFR-TKIs block the activation of downstream signaling in the EGFR pathway. ICIs alter the immune response and reverse the immunosuppressive microenvironment in *EGFR*-mutated ADC. TIME, tumor immune microenvironment environment; EGFR, epidermal growth factor receptor; TKIs, tyrosine kinase inhibitors; ADC, adenocarcinoma; MDSC, myeloid-derived suppressor cell; NK, natural killer; NKT, natural killer T; IDO, indoleamine 2, 3-dioxygenase; DC, dendritic cell; MHC, major histocompatibility complex; IFN-γ, interferon-gamma; TNF-α, tumor necrosis factor alpha; PFN, perforin; GzmB, Granzyme B; NSCLC, non-small cell lung cancer.

**Table 1:** Effects of common oncogenic driver mutations on TIME in lung ADC.

Oncogenes	Effects on TIME	Ref.
*EGFR*	Decreased mutation burden.	[109]
	Decreased PD-L1 expression, lower proportion of PD-L1^+^/CD8^+^ TIL, higher proportion of PD-L1-TIL.	[2,110]
	Inducing apoptosis of T cells.	[111]
	Lack of CD8^+^ T-cell infiltration, lower fractions of granzyme B^+^CD8^+^ T cells, lower CD8^+^ tissue-resident memory T cells.	[111,112]
	Enriched resting memory CD4^+^ T cell, lack of activated memory CD4^+^ T cells, decreased CXCL13-producing CD4^+^ T cells.	[112,113]
	Inducing Treg cells.	[114,115]
	Decreased B cells, lower expression of MHC-I and -II on B cells, decreased activation and cytotoxicity of cytotoxic NK cells, increased cytotoxicity of NKT cells.	[112]
	Less M2-TAMs, enhanced M2-like polarization and migration ability of TAMs, expansion of alveolar macrophages (AMs: lung cancer tissue-resident macrophages).	[116]
	Up-regulate MDSCs.	[98]
	Inducing the production of IDO by DCs, Higher proportions of CD1A^+^ DC and CD1C^+^ DC.	[117]
	Secreting exosomes harboring PD-L1, and PD-L1 in exosomes is resistant to PD-L1 inhibitors.	[118]
	Lower levels of human leukocyte antigen (HLA)-B expression in the presence of IFN-γ.	[119]
	Increased the serum levels of IL-10 and CCL2.	[120]
	High expression of CD73.	[120]
	Increased CCL5 and CCL22.	[121]
	Decreased IRF, CXCL10 and CXCL13.	[122]
*ALK*	Low TMB.	[2]
	Higher proportion of PD-L1-/TIL-tumors.	[113]
	Lack of CD8^+^T-cell infiltration.	[3]
	Enriched resting memory CD4+ T cell, lack of activated memory CD4^+^T cells.	[113]
*KRAS*	Higher PD-L1 expression, higher TIL.	[2]
*HER2*	Low TMB.	[28]
	Decreased PD-L1 expression.	[123]
*MET*	Higher CD4^+^ T-cell infiltration.	[124]

**Table 2:** TIME in lung ADC with oncogenic driver mutations after targeted therapy.

Targeted Inhibitors	Effects on TIME	Ref.
EGFR inhibitors	Increased CD8^+^ T cells.	[98]
	Reducing T cell apoptosis.	[125]
	Increasing IFN-γ secretion.	[120]
	Inhibition of M2-like polarization of macrophages.	[116]
	Decreased FoxP3^+^ Tregs, reducing the infiltration of Tregs.	[125]
	Induction of MHC-I and MHC-II molecules.	[126]
	Increased PD-L1 expression level of ≥50%.	[121]
	Higher mutation burden.	[121]
	Increased CXCL10.	[127]
	Increased infiltration of B cells.	[128]
	Enhanced cytotoxicity of NK cells.	[129]
ALK inhibitors	Increased CD8^+^ T cells, gamma-delta T cells.	[128]
	Increased T cell infiltration.	[106]
	Increased T cell activation and differentiation.	[128]
	Increased in the infiltration level of NK CD56^dim^ cells.	[128]
	Up-regulated HLA-I expression	[130]
KRAS inhibitors	Lower PD-L1 expression.	[131]
MET inhibitors	Higher 4-1BBL, OX40L, and CD70.	[132]
	Lower PD-L1 expression.	[132]

## 5. Novel Techniques in Characterization of Tumor Immune Phenotypes

Conventional technologies used for characterizing tumor immune phenotypes are limited by modest analysis parameters and large quantities of specimens required. Advances in various technologies, including spatial single-cell transcriptomic analysis, imaging mass cytometry, and quantitative multiplex imaging.

The spatial single-cell transcriptomic analysis is a technique used to study gene expression patterns within individual cells in a tissue sample while also preserving their spatial context. This technique combines in situ hybridization, imaging mass cytometry, and spatially resolved RNA sequencing, which allows researchers to map the location of cells within a tissue and analyze their gene expression profiles at the same time. Spatial transcriptomic profiling of lung tumors and brain metastases suggested that the tumor microenvironment of the brain is distinguished by diminished antigen presentation and impaired B/T cell activity, heightened neutrophils and M2-type macrophages, immature microglia, reactive astrocytes [133]. Additionally, it has been found an enrichment of CD163^+^CD33^+^PD-L1^+^ TAMs in tumors from hyper-progressor patients in comparison with patients not experiencing hyper-progression strongly suggests the spatial distribution of TAMs plays an important role in determining the outcome of patients with NSCLC treated with ICIs [134].

Imaging mass cytometry combines laser ablation to vaporize tissue samples and ionize the resulting particles, which are then analyzed using mass spectrometry to determine the presence and quantity of specific proteins or molecules in individual cells. Like spatial single-cell transcriptomic analysis, this method allows the analysis of up to 40 protein markers on a tissue section while preserving the tissue architecture and enables the in-situ characterization of tumor immune contexture. A recent study using imaging mass cytometry coupled with deep learning allows the prediction of recurrence with high accuracy with routine surgical resection (a 5-µm section of a single 1-mm^2^ core of formalin-fixed paraffin-embedded tumor tissue) [135].

Quantitative multiplex immunofluorescence image analysis is a powerful tool used to characterize tumor immune phenotypes by simultaneously detecting multiple cell markers in a single tissue sample. This technique allows for the identification of different cell populations and, most importantly, provides detailed information about the spatial distribution and abundance of different immune cell populations within the tumor microenvironment. The previously established Immunoscore analyzed only the expression of CD3 and CD8 by immunohistochemistry in the invasive margin and center of the tumor [136]. The densities of CD3^+^ and CD8^+^ T cells are scored on a scale from 0 to 4 based on the area occupied by the immune cells in the invasive margin and center of the tumor. However, with the advancement of multiplex immunofluorescence imaging, precise characterization of the immune landscape of the tumor or even quantitative analysis of the TIME is now feasible [137]. A study using quantitative multiplex immunofluorescence imaging showed CD8/PD-L1 or CD68/PD-L1 co-expression was associated with the efficacy of ICIs plus chemotherapy as first-line treatment in patients with advanced NSCLC [138].

## 6. Conclusions and Future Directions

Both targeted therapy and immunotherapy have shown promising therapeutic efficacy in defined groups of advanced-stage or locally advanced lung cancer patients. However, a combination of targeted therapy and immunotherapy did not show synergistic antitumor effects both in vitro and in clinical trials. Further characterization of TIME in oncogene-driven NSCLC suggested a generally weak immunogenic TIME. Innovative preclinical studies explored different entry points in TIME [47] to foster a better understanding of the fundamental mechanisms of combination therapy in the context of TIME. As described above, dynamic interactions and a variety of immune cells modulate the TIME, so it is necessary to evaluate the effects of ICI without limiting the focus on the interactions between T cells [139] and tumor cells [140]. With those novel techniques for detailed characterization of tumor immune phenotypes discussed above, together with further development of well-characterized preclinical models, this would offer a novel therapeutic intervention that would allow for comprehensive, precise and personalized immune-based therapeutic targeting the impact of imbalanced TIME in advanced NSCLC.
